# Preserving the vitality of teeth adjacent to a large radicular cyst in periapical microsurgery: a case report with 4-year follow-up

**DOI:** 10.1186/s12903-021-01738-2

**Published:** 2021-08-03

**Authors:** Ahmed Elhakim, Sunil Kim, Euiseong Kim, Alaa H. Elshazli

**Affiliations:** 1 Department of Endodontics, Faculty of Dentistry, Mansoura University, Mansoura, 35516 Egypt; 2 Microscope Center, Department of Conservative Dentistry and Oral Science Research Center, College of Dentistry, Yonsei University, Seoul, 03722 Republic of Korea

**Keywords:** Endodontic microsurgery, Neurovascular bundle, Pulp vitality, Radicular cyst, Sensibility test

## Abstract

**Background:**

Radicular cysts may enlarge considerably, cause extensive bone destruction, and jeopardize the integrity of the associated vital teeth. The different treatment approaches are aimed mainly at eliminating the cystic epithelial membrane while reducing the risk of injury to vital structures. Contrary to other treatment modalities, preapical surgery offers an unequivocal single occasion resolution for the patient. However, it has been associated with higher risk of collateral damages.

**Case presentation:**

A patient presented with a large radicular cyst originating from a maxillary lateral incisor. The adjacent central and canine teeth initially failed to exhibit responses to sensibility tests but showed signs of vitality. Microsurgical management was aimed at enucleating the cystic membrane while maintaining adjacent teeth vitality. Upon careful and controlled cyst enucleation under the dental operating microscope, the neurovascular bundle of one of the involved teeth was visualized and its integrity was maintained throughout the procedure.

**Results:**

The procedure was successful and follow up recalls revealed recovery of normal sensibility of tooth 11 and 13 with complete bone regeneration around their apices.

**Conclusion:**

Within the limitation of the present case report, we demonstrated that complete excision of large periapical cyst can be performed without sacrificing the vitality of the adjacent teeth, by preserving the integrity of their neurovascular supply through controlled microsurgical enucleation, and by a potential apical vascular repair ensuing unintended injury. Diagnosing the pulp vitality of non-offending teeth whose apices protrude into the cystic lumen is a complex process and can be misleading. Pressure from the growing cyst can inhibit vital teeth responses to neural-based sensibility tests leading to false negative results. Thus, in such cases, the use of blood perfusion-based vitality testing is recommended for correct initial diagnosis.

## Background

Radicular cyst is the most common cystic pathology of the jaws [1]. It arises as a sequela of inflammatory processes of the pulp and periodontium from the epithelial rests of Malassez and may enlarge to considerable sizes while causing extensive bone destruction [2].

Vitality of non-offending teeth, whose apices protrude into the cystic lumen and are overlapped by the cystic membrane, is a pathognomonic sign of radicular cysts. The slow expansion growth of the cystic membrane resorbs the bone but deflects other structures including major nerves and apical neurovascular bundles of adjacent teeth, compromising their integrity [3], and leading to confounding diagnosis [4, 5].

Management of periapical inflammatory cysts includes non-surgical endodontic treatment of the causative tooth, marsupialization, and surgical enucleation [2, 6]. Various reports identified extra radicular sources of irritations in cases of persistent radicular cysts [7, 8]. Thus, both the radicular space and the cyst itself should be considered as individual potential sources of morbidity and treated simultaneously. Decompression has been cited as successful conservative surgical approach for treating large odontogenic cysts [6]. However, demanding patients tasks, extended follow-up and the frequent second operation, make marsupialization less tolerable by patients. On the other hand, cyst enucleation by surgery precludes the prognostic uncertainty and patient’s discomfort associated with the other treatment modalities, since no pathological tissues remain after treatment and the follow-up recalls monitor the progress of healing rather than the regression of an existing disease process.

In surgical excision of an extensive radicular cyst, the operator may be faced with the dilemma of having to pre-emptively devitalize the healthy neighbouring teeth. The rationale for the prophylactic treatment is to prevent of future periodontitis, as devitalization is considered inevitable while enucleating the cystic membrane [5]. Nevertheless, due to the pivotal rule of the vital pulps in defence and repair and their inherent value as source of stem cells, all effort should be made to avoid unnecessary devitalization whenever possible [9].

Advancement in endodontic surgical armamentarium and magnification enhanced the clinician’s critical assessment of the surgical field and control over the procedure, improving the long-term outcomes [10, 11]. If the vitality of the surrounding teeth is closely regarded during the surgery by carefully excising the cystic membrane covering their apices without disrupting their neurovascular supply, the advantages of both the surgical and conservative cystic treatments can be combined while their drawbacks minimized.

This case report describes the surgical enucleation of a large maxillary radicular cyst aimed at preserving the vitality of the associated teeth and the results of long-term follow-up.

## Case presentation

A 41-year-old woman was referred to the endodontic department at Mansoura University, Mansoura, Egypt, for evaluation of a periapical lesion related to the roots of maxillary right anterior teeth. The lesion was discovered inadvertently during routine radiographic examination. The patient had been asymptomatic, and her main complaint was related to difficulties in mastication due to loss of multiple posterior teeth. Prior medical history was non-contributory.

**Fig. 1:**
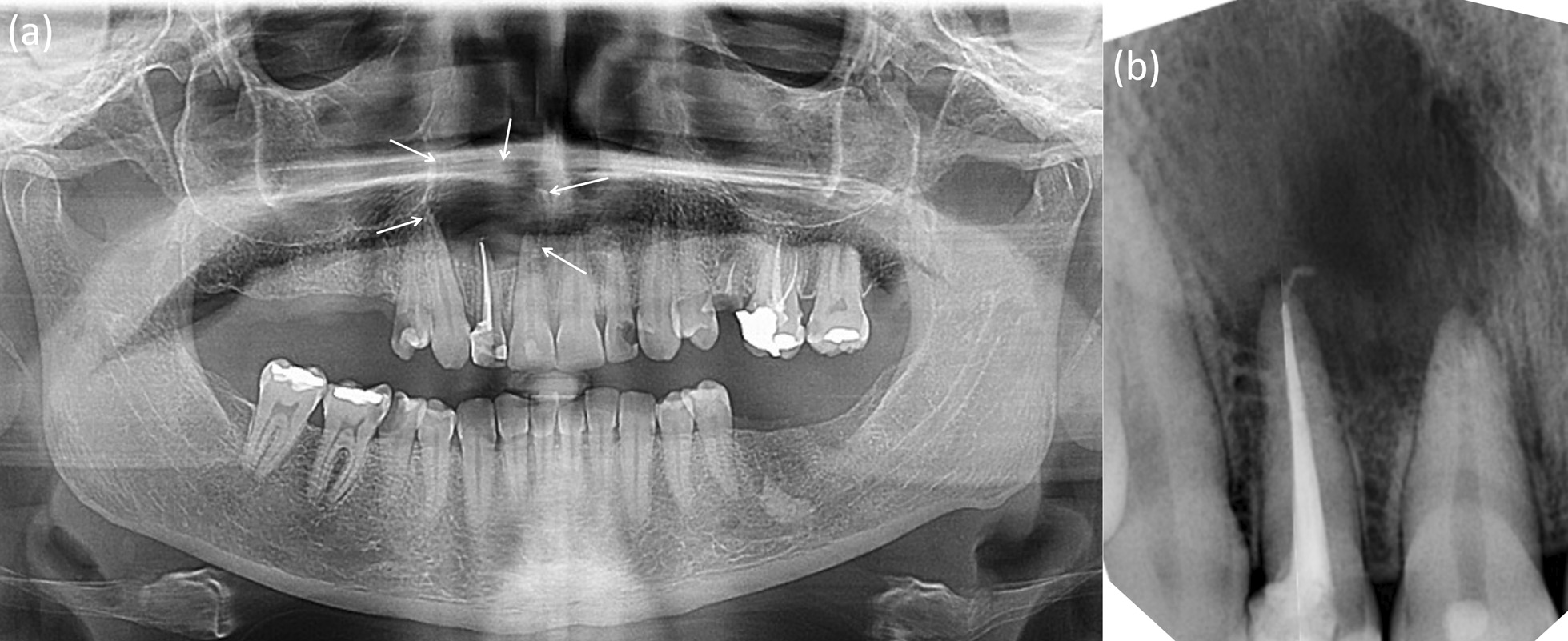
Radiographic x-ray taken after root canal treatment on tooth 12. **a** Panoramic radiograph showing radiolucency extending from tooth 13 to 11 (arrows) **b** Periapical radiograph. Note the normal pulpal morphologies of teeth 13 and 11

Clinical examination revealed a grossly decayed upper right lateral incisor (FDI tooth no. 12). The labial vestibular mucosa and the palate surrounding the tooth apex were compressible but not tender to palpation, indicating that they were undermined with no underlying cortical bone plates. Panoramic view radiography (Fig. 1) revealed a large well-defined radiolucent area extending from the distal of tooth 13 to the mesial of tooth 11 and resembling a “through-and-through” bone defect. Teeth 13 and 11 were sound and were not sensitive to percussion or palpation. However, they both failed to exhibit responses to repeated sensibility tests performed with an electric pulp tester (EPT) (Denjoy Dental Co, Ltd, Hunan, China) and Endo-Ice (1,1,1,2 tetrafluoroethane; Hygenic Corp, Akron, OH, USA) (contra laterals as control) and were deemed nonvital. None of the examined teeth had any periodontal symptoms. Clinical and radiographic findings were consistent with a representation of a radicular cyst originating from tooth 12. After critical appraisal of the clinical and radiographic findings and discussion with the patient, surgical enucleation was elected to manage the periapical lesion. The patient was scheduled for root canal treatment of the involved teeth in preparation for the surgical intervention.

After informed consent and without anaesthesia, tooth 12 was accessed under rubber dam and emitted pungent odour revealing a necrotic pulp. The root canal was cleaned, and shaped while being irrigated with a frequently refreshed 5.25% sodium hypochlorite. The canal did not discharge cystic fluid despite frequent encouragement of patency file through the apex and a final irrigant activation by manual dynamic agitation. Following dryness, the canal was filled with guttapercha cones and TotalFill biocermaic sealer (FKG Dentaire SA, La Chaux-de-Fonds, Switzerland) (Fig. 1b). When access cavity was initiated for root canal treatment on tooth 11, which was diagnosed with pulpal necrosis, the patient unexpectedly exhibited sudden pain when the bur almost breached the pulpal space. The initial diagnosis of nonvitality for the central incisor and the canine was reconsidered, as both teeth were sound. The cavity was restored, and a decision was taken not to proceed with the planned root canal therapy.

**Fig. 2:**
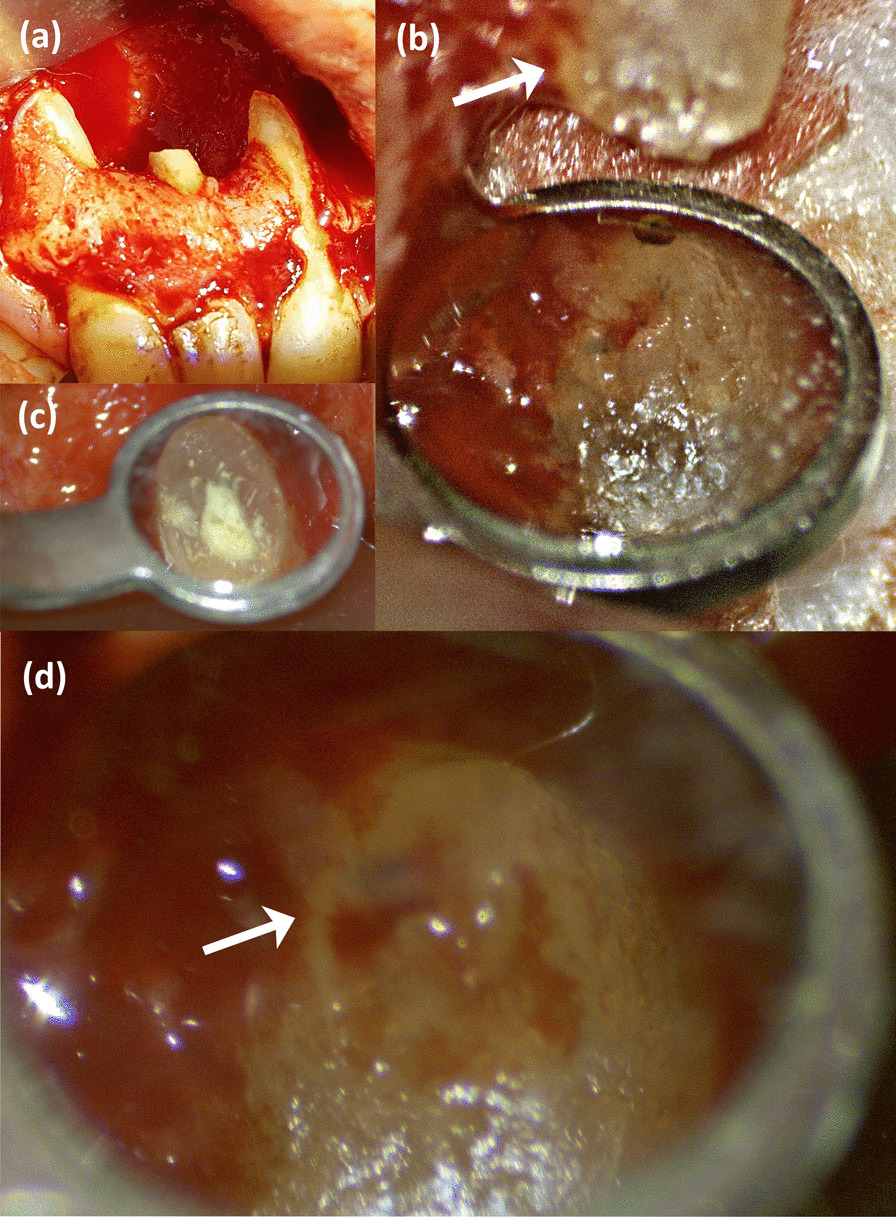
Intraoperative photographs. **a** The bone cavity after root end resection and cyst enucleation. **b** Tooth 11 root apex and the innervating neurovascular bundle visualized after cyst enucleation. The arrow indicates the neurovascular bundle course on the mesial root surface. **c** Rechecking the integrity of the neurovascular bundle entering the apical foramen (Arrow) of tooth 11 prior to flap repositioning

All surgical procedures were performed using a surgical microscope (M320, Leica Microsystems, Heerbrugg, Switzerland), except the incisions, flap elevation, and suturing. 2% lidocaine (with 1:100,000 epinephrine) (Alexandria Co., Alexandria, Egypt) was administered and a full thickness buccal flap was elevated to reveal the cystic lining covering an extensive cortical bone defect. No osteotomy was needed. After identification of tooth 12 root, a 3 mm apical part was cut under copious irrigation with sterile saline. The cystic lining was completely removed by careful dissection starting from the middle craniofacial defect edge and advancing in anteroposterior directions. The cystic membrane was peeled in downward motion along the osseous wall rather than being pulled away from it. Whenever needed, releasing incisions were made in the cystic membrane to facilitate its removal. Following enucleation, the cavity was examined, revealing expansion of the cyst buccally, palatally as well as in the direction of the nasal floor, exposing the nasal mucosa (Fig. 2a).

The neurovascular bundle of tooth 11 was observed running from the apical foramen mesially toward the caudomedial defect wall (Fig. 2b). Due to the geometry of the defect in relation to tooth 13 root, similar observation was not possible. Thus, all subsequent procedures were done cautiously to avoid damages. The resected root surface was inspected, prepared, and then filled with pro root MTA (Dentsply/Tulsa Dental, Tulsa, OK, USA) (Fig. 2c). Prior to flap repositioning, the neurovascular bundle of tooth 11 was rechecked again to ensure its integrity (Fig. 2d). Primary mucoperiosteal closure on solid margins was accomplished with no releasing incisions and the flap was sutured with 4–0 polypropylene suture (International Sutures, Shrkia, Egypt). Postoperative instructions included a twice daily rinse with 0.12% chlorhexidine gluconate (Peridex, 3M ESPE, St Paul, MN, USA) for 1 week. The patient returned to remove the sutures with uneventful soft tissue healing and no reported complications. The histopathologic examination confirmed the initial diagnosis of an inflammatory radicular cyst.

**Fig. 3:**
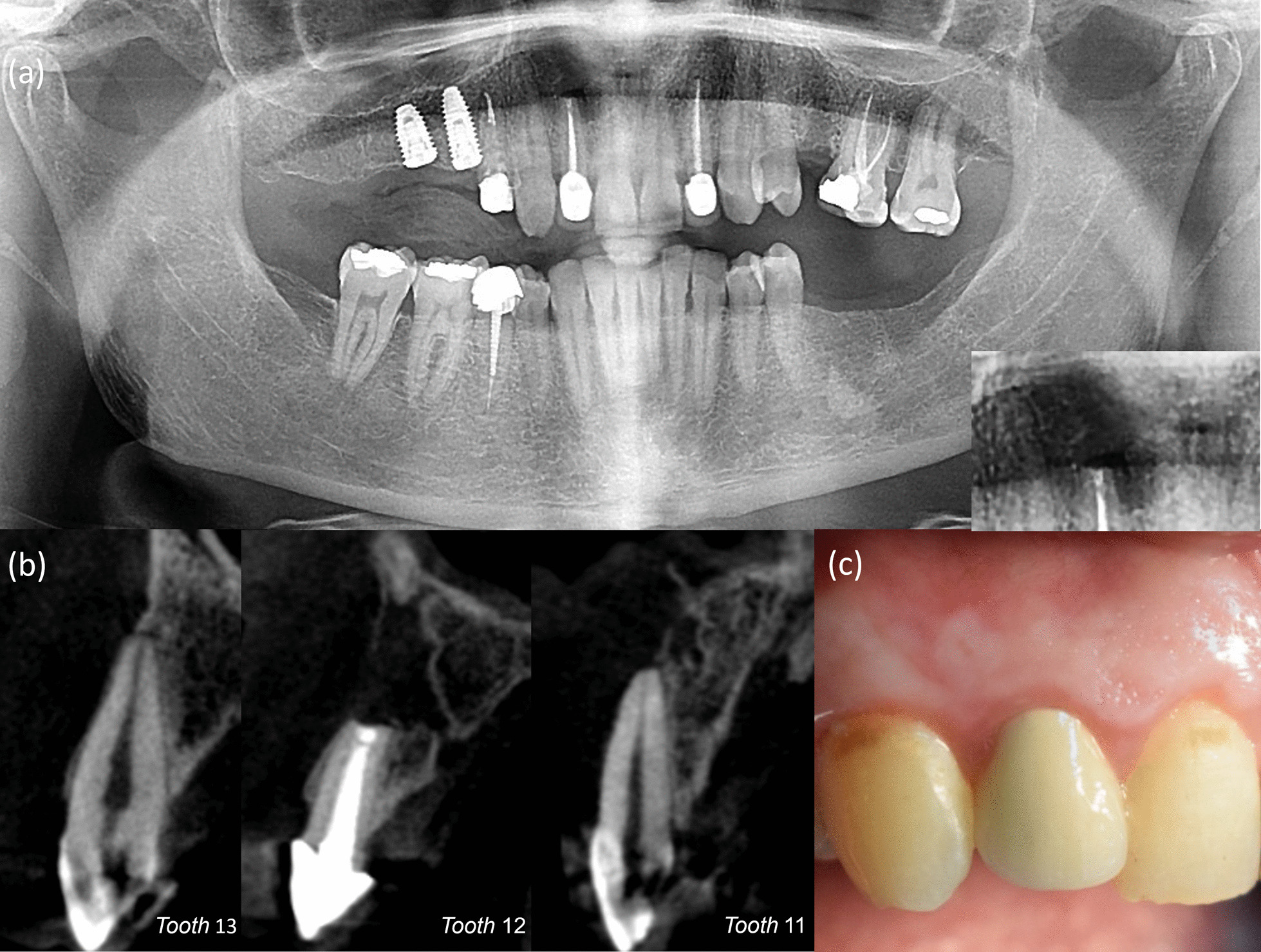
One-year follow-up. **a** Panoramic radiograph demonstrating angular relation of regenerated bone to the resected root surface. **b** Cone-beam computed tomography images. Sagittal plane on tooth 13, 12 and 11. Tooth 13 demonstrates near obliteration of coronal pulp. **c** Clinical photo. Soft tissue healing at the site of surgery

At the 1-year follow-up, clinical examination revealed that teeth 13 and 11 regained normal responsiveness to EPT and cold sensibility tests. Panoramic radiograph demonstrated reduction in the defect size with partial bone fill around apices of teeth 13, 11 and up to the apicoectomy level of tooth 12 root (Fig. 3a). The 2-dimensional (2D) radiographic healing was judged incomplete by two independent and calibrated reviewers as per Molven’s criteria [12]. However, it was in disagreement with the 3-dimensional (3D) healing outcome on the cone-beam computed tomography (CBCT) (Fig. 3b), as it was considered uncertain according to modified PENN 3D criteria. [13] As the patient reported absence of any discomfort related to the treated teeth or surgical site since the operation with normal soft tissue (Fig. 3c), a second recall appointment was set at the 4-year mark following the operation.

**Fig. 4:**
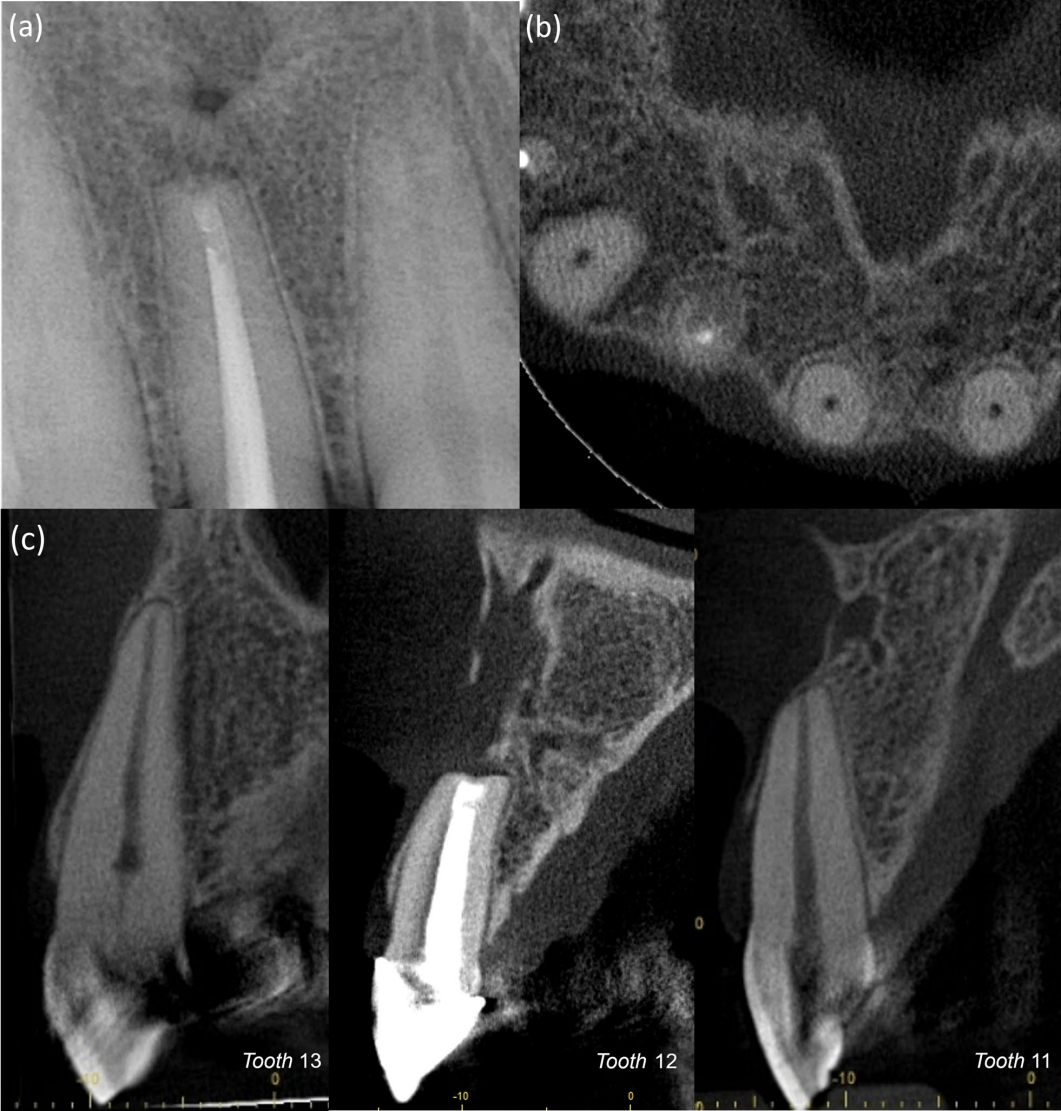
Four years after the surgery. **a** Periapical radiograph. **b** Cone-beam computed tomography images. Axial plane at the resected root level. **c** Sagittal plane on tooth 13, 12 and 11

Teeth 13 and 11 remained responsive to sensibility tests with normal signs of vitality four years after surgery. Periapical radiograph and CBCT demonstrated continued and significant reduction in the lesion size, indicating a change to an agreement between both the 2D and 3D radiographic healing criteria toward incomplete (limited) healing by scar tissue (Fig. 4). Complete regeneration of bone and periodontium around the apices of teeth 13, 11 was evident.

## Discussion

Traditionally, vigorous removal of the cystic pathological tissue in preapical surgery was advocated to rapidly establish haemostasis and reduce the surgical operation time [14]. In our case, a meticulous and controlled excision of the cystic membrane was performed under magnification by the DOM. This allowed us to preserve tooth 11 neurovascular bundle and the exposed nasal mucosa. While the detection of the neurovascular supply of the tooth after enucleation of the cyst epithelium (Fig. 2b) and before flap repositioning (Fig. 2d), allowed us to accurately predict the prognosis of vitality of one of the two vital teeth included in the cystic cavity. Response to sensibility tests in follow-up recalls and absence of periapical radiolucency, resorption, and coronal discoloration are ancillary evidence that support the maintenance of tooth 11 and 13 to vitality.

Additionally, tooth 13 demonstrated partial calcific obliteration (PCO) at the follow-up investigations. In absence of other conditions, PCO is a sign of vascular repair, indicating a possible injury to tooth 13 apical vasculature while handling the cystic epithelium [15]. The deposited dentin is considered reparative, as it’s formed by newly differentiated mesenchymal cells following odontoblasts degeneration secondary to the injury [16]. We noticed a near obliteration of coronal pulpal space at the 1-year follow-up radiographs (Fig. 3b). However, over the course of the following 3 years, the rate of calcification was significantly reduced with only a slight narrowing of the radicular canal (Fig. 4c). The initial pronounced calcification of the coronal pulp may be attributed to the tendency of inflammation to localize coronally after injury [16]. While reinstatement of the neural regulatory control over the newly differentiated odontoblast and reversal of hypoxic conditions in the pulp might have been the reasons of the limitation of calcification [17, 18].

Lundeberg and Cvek study [19] on PCO of traumatized teeth concluded that pre-emptive endodontic treatment is not warranted, based on the histological status of pulps undergoing calcification. Anderson et al. [15] reported that secondary pulpal necrosis happened only to 1% of teeth exhibiting PCO during a 1–10-year follow-up study, while Robertson et al. [20] calculated a 20-year pulp survival rate to be 84% for permanent teeth with calcified pulps secondary to dental trauma. As tooth 13 remains vital, no further management is needed.

Guided tissue regeneration techniques utilized in periapical surgeries, are intended to improve the healing outcome in large osseous defects with resorbed cortices by placing a membrane covering an osteogenic material packed in the bone cavity [21]. Although a membrane barrier might have been beneficial in this case, packing the grafting material could have jeopardized the integrity of the vital teeth neurovascular bundles and limited their repair potential. This sighting adds a new factor to consider for the use of regenerative techniques in periapical surgeries.

Teeth 13 and 11 lacked positive responses to initial sensibility tests done by EPT and cold test but regained and maintained normal responsiveness following treatment. This could be attributed to compression neuropathy caused by the expanding cyst [22]. Positive pressure is considered a key regulator of cystic growth [23]. It has been linked to initiation of bone resorptive mechanisms and to alterations in the physiology of the surrounding structures [4, 24].

In neurological studies, applied pressures of 50 mm Hg and 60 mm Hg completely inhibited axonal transport function and interneural blood flow in rabbit vagus and tibial nerves, respectively [25, 26]. In addition, a pressure value between 40 mm Hg and 50 mm Hg is considered a critical threshold for the disruption of peripheral nerves conduction and response to cutaneous sensibility tests in healthy patients [22, 27]. Those values lie well within the + 47 and + 70 mm Hg measured means of intracystic pressure for radicular cysts [28, 29], and a proposed minimum continuous pressure of 51 mm Hg necessary to induce bone resorption in the hard palate, based on animal experiment on rats [30]. Ricuccui et al. [5] reported a similar incident when two vital premolars, whose apices were compressed by a large radicular cyst, only gave faint responses to cold sensibility tests and did not respond to EPT, while other reports showcased the recovery of inferior alveolar nerve sensory function following decompression of large cystic lesions and illustrated improved responsiveness of the innervated teeth [4, 24].

Additionally, the patient sudden painful reaction while accessing tooth 11 further supports the pressure hypothesis. The larger myelinated A- type nerve fibres are pressure and hypoxia sensitive compared with C- type nerve fibres [31]. While the pulpal response to electrical and cold sensibility tests mediated by A- fibres might have been suppressed, the heat build-up and mechanical stimulation by the cutting bur caused a latent C- fibre pain response [32]. Interestingly, at multiple occasions when similar cases of large cysts and cystic like lesions were presented to our clinic, adjacent vital pulps were accessed without anaesthesia, demonstrating complete lack of sensation, especially in cases of secondary infections.

## Conclusion

Within the limitation of the present case report, we demonstrated that complete excision of large periapical cyst can be performed without sacrificing the vitality of the adjacent teeth, by preserving the integrity of their neurovascular supply through controlled microsurgical enucleation, and by a potential apical vascular repair ensuing unintended injury. Diagnosing the pulp vitality of non-offending teeth whose apices protrude into the cystic lumen is a complex process and can be misleading. Pressure from the growing cyst can inhibit vital teeth responses to neural-based sensibility tests leading to false negative results. Thus, in such cases, the use of blood perfusion-based vitality testing is recommended for correct initial diagnosis.

## Data Availability

All data generated or analysed during this case are included in this published article.

## Abbreviations

EPT: Electric pulp tester
CBCT: Cone-beam computed tomography
DOM: Dental operating microscope
PCO: Partial calcific obliteration
